# Supplementary material for: Nutrition Management in Older Adults with Diabetes: A Review on the Importance of Shifting Prevention Strategies from Metabolic Syndrome to Frailty
Source: Nutrients. 2020 Nov 1;12(11):3367. doi: 10.3390/nu12113367 (PMC7693664; doi:10.3390/nu12113367)
Supplement: Supplementary file 1 [file nutrients-12-03367-s001.pdf]

Supplementary Table S1. Evidences of associations of nutritional status and mortality, frailty, sarcopenia and cognitive impairment

Obesity and mortality, frailty, cognitive impairment

Authors, Ref	Design	Subjects	Period	Intervention, exposure	Outcome (Evaluation Tests)	Results
Martínez-González, M.A. et al.[75]	Prospective Cohort	7447 Spanish older adults at high cardiovascular risk (55-80 y.o.)	4.8 years (median)	BMI, waist-to-height ratio (WHtR), waist circumference (WC)	all-cause mortality	High BMI was not associated with mortality. HR for mortality in high WHtR (≥0.70) and high WC (≥110cm) were 1.55(95%CI 1.06-2.26), 1.57 (1.19-2.08)
Tanaka, S. et al.[77]	Prospective Cohort	2 cohorts of 2620 Japanese diabetic patients (≧40y.o.)	4 years	BMI	All-cause mortality	HR for mortality was high in BMI<18.5kg/m2 group vs 22.5-24.9kg/m2 group (HR 2.58, 95% CI 1.38-4.84)
Schaap, L.A. et al.[79]	Meta-analysis	Meta-analysis of 50 observational studies in older adults (≧65y.o.)	1.5-13 years	body composition (BMI, waist circumference etc.) and muscle strength measures	Functional decline (self-reported ADL etc.)	BMI ≥30 was associated with functional decline (OR = 1.60, 95% CI): 1.43-1.80)
Nam, G.E. et al.[27]	Prospective Cohort	167,876 new-onset Korean type 2 diabetic patients (≧40y.o.)	3.5 years	Baseline BMI, Body weight change	Incident of dementia (ICD-10-CM code)	Baseline BMI was inversely associated with the risk of all cause dementia and Alzheimer's disease (p<0.001).

Metabolic syndrome and mortality, frailty, cognitive impairment

Authors, Ref	Design	Subjects	Period	Intervention, exposure	Outcome (Evaluation Tests)	Results
Hillier, T.A. et al.[87]	Prospective Cohort	9,677 community-dwelling older women in US (≧65y.o.)	12.2 years	Metabolic syndrome and diabetic state	Total, CHD, CVD mortality	Among older women with diabetes, the metabolic syndrome (diabetes, obesity, and hypertension) increased the HR (2-3 folds) of total and CHD/CVD mortality
Monami, M. et al.[88]	Prospective Cohort	1716 Italian type 2 diabetic outpatient(≧(28-96y.o.)	4.7 years	Metabolic syndrome (NCEP-ATP III or IDF criteria)	CVD mortality	CVD mortality was higher in subjects with NCEP-defined MetS vs without MetS≤70 years, whereas it was not different over age 70 years

Laudisio ,A. et al.[92]	Prospective Cohort	1155 Italian population-based older adults(≧ 65y.o.)	3 years	Metabolic syndrome (NCEP-ATP III criteria)	ADL disability (Katz's ADLs, the Lawton and Brody (IADL))	In subjects ≥74 years, probability of ADLs disability reduced (OR = 0.33, 95% CI = 0.14–0.77), whereas probability of IADL disability was not different
Yaffe, K. et al.[93]	Prospective Cohort	1624 US older Latinos (≧ 60y.o.)	3 years	Metabolic syndrome (NCEP-ATP III criteria)	Change in 3MS and the Delayed Word-List Recall (DelRec) scores	Subjects with Mets showed worse 3-year change on 3MS (P=.04) and DelRec (P=.03).
Komulainen, P. et al.[94]	Prospective Cohort	101 population-based older women in Finland (60-70y.o.)	12 years	Metabolic syndrome (NCEP criteria)	Global cognitive function, memory, cognitive speed	Subjects with Mets had 4.27 times (95%CI 1.02-17.90) higher risk of poor memory
Sarcopenic obesity and mortality, frailty, fall, fracture						
Authors, Ref	Design	Subjects	Period	Intervention, exposure	Outcome (Evaluation Tests)	Results
Atkins, J.L. et al.[98]	Prospective Cohort	4252 population-based older men in UK(60-79y.o.)	11.3 years	Sarcopenia (midarm muscle circumference), obesity (waist circumference)	CVD and all-cause mortality	All-cause mortality risk was higher in sarcopenic and obese men, but highest in sarcopenic obese (HR1.72, 95%CI 1.35–2.18)
Hirani, V. et al.[99]	Prospective Cohort	1705 community-dwelling Australian older men (≥70 y.o.)	5 years	Low muscle mass (appendicular lean mass/BMI), obesity (≥30% fat)	Frailty (CHS, SOF frailty index), ADL disability (Katz), IADL disability	Sarcopenic obesity (as well as low muscle mass) was associated with higher incidence of frailty (OR 2.00, 95%CI 1.42-2.82), ADL disability (1.58, 1.12-2.24), IADL disability (1.36, 1.05-1.76).
Scott, D. et al.[100]	Prospective Cohort	1486 community-dwelling Australian community-dwelling older men (≥70 y.o.)	5 years	Sarcopenia (EWGSOP or FNIH), obesity (≥30% fat)	Change in hip BMD, 2-year fall rate	Sarcopenic obesity (defined EWGSOP criteria) was associated higher incidence of 2-year fall (Incidence rate ratio 1.66, 95%CI 1.16-2.37)
Scott, D. et al.[101]	Prospective Cohort	1089 community-dwelling Australian community-dwelling older adults (mean 62 y.o.)	5.1 years	Sarcopenia, dynapenia (lowest tertile for appendicular lean mass, lower-limb strength), obesity	BMD, fracture rate	Sarcopenic obese men had higher non-vertebral fracture rate vs non-sarcopenic non-obese (incidence rate ratio 3.0, 95 % CI 1.7-5.5), and vs obese alone (3.6, 1.7-7.4).

				(highest sex-specific tertile for total fat)		

Malnutrition and mortality, frailty, cognitive impairment

Authors, Ref	Design	Subjects	Period	Intervention, exposure	Outcome (Evaluation Tests)	Results
Liu, G.X. et al.[103]	Prospective Cohort	302 Chinese older diabetic patients (≧65y.o.)	2.4-2.8 years	Malnutrition, at risk of malnutrition (MNA)	length of hospital stay, the number of re-hospitalizations in 1 year and mortality	Malnourished patients had longer hospital stays (P = 0.003) and higher mortality rates (P < 0.001)
Turnbull, P.J. et al.[105]	Case-control	70 UK Community-dwelling older adults (35 diabetes)		MNA, anthropometric measurements,	Barthel Index, Nottingham Extended ADL score, handgrip strength.	Diabetic subjects had lower MNA. MNA scores were correlated with Barthel Index, Nottingham Extended ADL score, handgrip (all p<0.01).
Malara, A. et al.[107]	Prospective Cohort	174 Italian community-dwelling older adults (Average 74 y.o.)	6 months	Nutritional state (MNA)	cognitive performance (MMSE), functional state (Barthel index, ADL)	Baseline MNA and MMSE were associated (**P**<0.001). There was a strong correlation between cognitive deterioration and worsening of nutritional state (**P**<0.005)

Body weight change and mortality, frailty, cognitive impairment

Authors, Ref	Design	Subjects	Period	Intervention, exposure	Outcome (Evaluation Tests)	Results
Lee, AK. et al.[112]	Secondary analysis of RCT	10,081 patients with diabetes	Exposure 2 year, and median follow-up of 8.4 years	Weight loss or gain during the exposure period	Macro/micro-vascular events, CVD mortality, all-cause mortality	Subjects with >10% weight loss had higher risk for macrovascular events (HR 1.75, 95%CI 1.26-2.44), CVD mortality (2.76, 1.87-4.09), all-cause mortality (2.79, 2.10-3.71) irrespective of age category (< or ≥65 y.o.)
Nam, G.E. et al.[27]	Prospective Cohort	167,876 new-onset Korean type 2 diabetic patients (≧ 40y.o.)	3.5 years	Baseline BMI, Body weight change	Incident of dementia (ICD-10-CM code)	Subjects with ≥10% weight gain had higher risk for incidence of dementia (HR 1.38, 95%CI 1.08-1.76). Subjects with ≥10% weight loss also had higher risk for incidence of dementia (1.34, 1.11-1.63) and AD (1.26, 1.01-1.59).

Houston, D.K. et al.[113]	RCT (including post-intervention period)	Overweight and obese (BMI ≥ 25 kg/m2) middle-aged and older type 2 diabetic patients, the Look AHEAD Study (45-76 y.o.)	11 years (intervention) plus 1.5 years (after intervention)	intensive lifestyle intervention (ILI; weight loss through caloric restriction and increased physical activity) vs diabetes support and education (DSE)	Gait speed, expanded SPPB, grip strength	ILI group had low risk for slow gait speed (OR 0.84, 95%CI 0.71-0.99). ILI group had faster gait speed and higher SPPB score

BMI, body mass index; ADL, activities of daily living; CHD, coronary heart disease; CVD, cardiovascular disease; NCEP-ATP III, National Cholesterol Education Program-Adult Treatment Panel III; IDF, International Diabetes Federation; CHS, Cardiovascular Health Study; SOF, Study of Osteoporotic Fracture; IADL, instrumental activities of daily living; EWGSOP, European Working Group on Sarcopenia in Older People; FNIH, Foundation for the National Institutes of Health; BMD, bone mineral density; MNA, Mini Nutritional Assessment; MMSE, mini-mental state examination; SPPB, Short Physical Performance Battery

Supplementary Table S2. Evidences of associations of energy, macro/micro-nutrient intake, dietary pattern and mortality, frailty, sarcopenia and cognitive impairment

Energy intake and frailty, mortality

Authors, Ref	Design	Subjects	Period	Intervention, exposure	Outcome (Evaluation Tests)	Results
Omura et al.[116]	Prospective Cohort	756 Japanese older diabetic patients (≥65y.o.)	6 years	Energy intake (FFQ)	mortality	HR for mortality was high in Q1 (≤24.85 kcal/kgBW) (HR 3.83, 95% CI 1.62-9.09) vs Q3 (29.74-34.78 kcal/kgBW)
Schoufour, J.D. et al.[120]	Cross-sectional	2504 population-based cohort, middle-old aged adults, the Rotterdam Study (≥45y.o.)		Energy intake (FFQ)	Incidence of frailty (CHS criteria)	The risk of frailty decreased approximately by 5% with each 418.4 kJ (100 kcal) increase in total energy intake

Protein intake and frailty, sarcopenia, cognitive impairment, mortality

Authors, Ref	Design	Subjects	Period	Intervention, exposure	Outcome (Evaluation Tests)	Results
Yamaoka, T. et al.[131]	Prospective Cohort	2 cohorts of 2494 Japanese diabetic patients (≥40y.o.)	6 years	Protein intake (FFQ)	Mortality	Protein intake <0.92g/kg BW was associated with a higher risk of mortality (HR = 2.26, 95% CI= 1.34-3.82) vs ≥1.41g/kg BW group. In patients ≥75 years, this association remained after adjustment of the total energy intake
Levine, M.E. et al.[179]	Prospective Cohort	population-based US cohort (≥50 y.o.)	18 years	percent of calorie intake from protein high (≥20%), moderate(10-19%), low (<10%)	overall / CVD/ cancer / diabetes-caused mortality	In those ages 50-65, high protein group had increased risk of overall (HR 1.74, 95%CI 1.02-2.97) and cancer mortality (4.33, 1.96-9.56) vs low group, whereas in those ages ≥66, both risk reduced (overall 0.78, 0.62-0.99; cancer 0.40, 0.23-0.71).
Isanejad, M. et al.[122]	Prospective Cohort	440 women, population-based cohort, (65-72 y.o.), Finland	3 years	Protein intake (3-day food record)	Incidence of prefrailty, frailty (CHS criteria)	Protein intake ≥ 1.1 g/kg BW was associated with a lower risk of prefrailty (OR = 0.45, 95% CI= 0.01-0.73) and frailty (OR = 0.09, 0.01-0.75)

Otsuka, R. et al.[123]	Prospective Cohort	283 Japanese population-based cohort, (65-86 y.o.)	2 years	Protein intake (3-day food record)	Incidence of prefrailty, frailty (CHS criteria)	Higher protein intake was negatively associated with frailty development (OR=0.72, 95% CI 0.53-0.97) /1SD=16 g)
Coelho-Júnior, H.J. et al.[124]	Meta-analysis	Meta-analysis of 7 observational studies in community-dwelling older adults (≥60y.o.)		Protein intake (various, 24-h dietary recall, 3-day dietary record etc.)	lower limb physical function (knee extensor strength, SPPB, and walking speed)	a very high (≥1.2 g/kg/day) and high protein intake (≥1.0 g/kg/day) are associated with better lower-limb physical performance, when compared to low protein (<0.80 g/kg/day) intake
Tieland, M. et al.[125]	Meta-analysis	Meta-analysis of 8 RCTs of elderly people (Mean≥65y.o.)	84-730 days	protein or amino acid supplementation	muscle mass and strength (lean body mass, leg press strength, leg extension strength, handgrip strength	protein or amino acid supplementation could not improve muscle mass or strength
Hanach, N.I. et al.[126]	Meta-analysis	Meta-analysis of 11 RCTs of aged between 61 and 81 y.o., with and without sarcopenia	12 weeks-2 years	dairy protein supplementation or a protein-based dairy product	appendicular muscle mass, muscle strength of handgrip and leg press, SPPB	Dairy protein significantly increased appendicular muscle mass (0.13 kg; 95% CI: 0.01-0.26), but did not improve handgrip or leg press. The effect on the SPPB was inconclusive.
Park, Y. et al.[127]	RCT	120 Korean older adults with ≥1 CHS criteria and ≤23.5 MNA score (70-85 y.o.)	12 weeks	Protein intake (0.8, 1.2, 1.5 g/kgBW/d)	appendicular skeletal muscle mass (ASM), Frailty (CHS), gait speed	1.5 g/kgBW/d group showed higher increase of ASM (0.52 vs 0.08 kg) and gait speed (0.09 vs 0.04 m/s) vs 0.8 g/kgBW/d group
Rahi, B. et al.[129]	Prospective Cohort	172 Canadian older diabetic adults (mean 75 y.o.)	3 years	Protein and energy intake	Change in functional capacity (Système de Mesure de l'Autonomie Fonctionnelle)	Adequate protein (≥1 g/kg BW) in women was associated with lesser functional capacity decline (p=0.029), whereas adequate energy intake (≥30kcal/kg BW)was not.
Roberts, R.O. et al.[133]	Prospective Cohort	937 population-based US nondemended subjects (median 79.5 y.o.)	3.7 years (median)	total daily caloric and macronutrient intakes (%) (FFQ)	Incidence of MCI and dementia (CDR, DSMIV)	The risk of MCI or dementia was reduced in subjects with high protein (p for trend 0.03) and fat (0.03) intake, whereas increased in high carbohydrate intake.
Vitamin D and sarcopenia, cognitive impairment						

Authors, Ref	Design	Subjects	Period	Intervention, exposure	Outcome (Evaluation Tests)	Results
Bauer, J.M. et al.[144]	RCT	380 sarcopenic primarily independent-living older adults, Europe, multicenter (≥65y.o.)	13 weeks	vitamin D and leucine-enriched whey protein nutritional supplement	handgrip strength, SPPB, chair-stand test, gait speed, balance score, appendicular muscle mass	The intervention group improved more in the chair-stand test and appendicular muscle mass
Bo, Y. et al.[145]	RCT	60 Chinese sarcopenic older adult	6 months	nutritional supplement containing whey protein, vitamin D and E	Relative skeletal mass index (RSMI), handgrip strength, 6-m gait speed, chair stand test, timed-up-and-go test, QOL	The intervention group improved RSMI, handgrip strength and QOL
Goodwill, A.M. et al.[146]	Meta-analysis	Meta-analysis of 26 (cross-sectional 12) observational studies in midlife and older adults without a diagnosis of dementia	4 months-13.4 years (longitudinal studies)	Blood vitamin D level	Various neurocognitive assessment scores (MMSE, TMT, etc.)	Low vitamin D was associated with worse cognitive performance (OR = 1.24, 95% CI 1.14-1.35) and cognitive decline (OR = 1.26, 1.09-1.23)
Goodwill, A.M. et al.[146]	Meta-analysis	Meta-analysis of 3 RCTs in midlife and older adults without a diagnosis of dementia (>18 yrs)	4 weeks -6 months	Supplementation of VD (oral or injection)	Various neurocognitive assessment scores (CDT etc.)	Vitamin D supplementation showed no benefit on cognition

Other vitamins and frailty, cognitive impairment

Authors, Ref	Design	Subjects	Period	Intervention, exposure	Outcome (Evaluation Tests)	Results
Balboa-Castillo, T. et al.[148]	Prospective Cohort	1,643 Spanish community-dwelling older adults (≥65y.o.)	3.5 years	10 vitamins (VA, thiamine, riboflavin, niacin, vitamins B6, B12, C, D, E and folates) lowest vs highest tertile of intake	Incidence of frailty (CHS criteria)	OR of frailty incidence was 2.80 (95% CI 1.38-5.67) for VB6, 1.65 (0.93-2.95) for VC, 1.93 (0.99-3.83) for VE and 2.34 (1.21-4.52) for folates

Forbes, S.C. et al.[153]	Meta-analysis	Meta-analysis of 13 RCTs of mid- older adults with normal cognition or MCI (≥40y.o.)	≥3 months	Omega-3 fatty acids, B vitamins, and vitamin E supplementation	Change in various cognitive test scores (MMSE, CANTAB, etc.)	Omega-3 fatty acids, B vitamins, and vitamin E supplementation did not affect cognition
Suh, S.W. et al.[154]	Meta-analysis	Meta-analysis of 38 RCTs of non-demended mid-older adults (≥40y.o.)	≥3 months	supplementation of B vitamins, antioxidant vitamins (C, A, E) or VD	Change in various test scores for global cognition (MMSE, CVLT, etc.)	B vitamins was beneficial for global cognitions (standardized mean difference (SMD) −0.18, (95% CI −0.30 to −0.06) and episodic memory (SMD −0.09, CI −0.15 to −0.04)
Araki, A. et al.[28]	Prospective Cohort	237 Japanese older diabetic patients (≥65y.o.)	6 years	Intakes of various food and nutrients (FFQ)	Cognitive decline (≥2-point decrease in MMSE)	low intake levels of carotene, vitamin B_2, pantothenate, calcium, and green vegetables were associated with cognitive decline ($p<0.05$)

Fatty acid (FA) and frailty, cognitive impairment

Authors, Ref	Design	Subjects	Period	Intervention, exposure	Outcome (Evaluation Tests)	Results
Jayanama, K. et al.[161]	Cross-sectional	4062 US population-based cohort, (≥50y.o.)		Intakes of 29 dietary FA	Change in frailty index, mortality	Higher intake of SFA intake was associated with higher frailty and mortality. Higher intake of PUFA and ω-3FA intake was associated with lower mortality.
Zhang, X W. et al.[165]	Meta-analysis	Meta-analysis of 6 RCTs	3-40 months	Intake of ω-3 FA (DHA+EPA)	Change in MMSE score	ω-3FA decreased the rate of decline in MMSE score (weighted mean difference =0.15, 95% CI 0.05-0.25)
Forbes, S.C. et al.[153]	Meta-analysis	Meta-analysis of 24 RCTs of mid- older adults with normal cognition or MCI (≥40y.o.)	≧3 months	Omega-3 fatty acids, B vitamins, and vitamin E supplementation	Change in various cognitive test scores (MMSE, CANTAB, etc.)	Omega-3 fatty acids, B vitamins, and vitamin E supplementation did not affect cognition
Brainard, J.S. et al.[166]	Meta-analysis	Meta-analysis of 38 RCTs of non-demended adults (≥18y.o.)	≧24 weeks	Food or supplements which increase ω-3, ω-6, total PUFA	new diagnosis of cognitive decline, global cognition (MMSE), executive function, processing speed, memory	Long chainω-3 has no effects in new cognitive illness (RR0.98, 95% CI 0.87-1.10) or cognitive impairment (RR0,99, 0.92-1.06) and very little

						effect in MMSE score (MD 0.10, 0.03-0.16). Effects ofω-6 and total PUFA were unclear.

Mediterranean diet, helthy diet pattern and mortality, frailty, cognitive impairment

Authors, Ref	Design	Subjects	Period	Intervention, exposure	Outcome (Evaluation Tests)	Results
Iimuro, S. et al.[180]	Prospective Cohort	912 Japanese older diabetic patients (≥65y.o.)	6 years	Dietary pattern (healthy, greasy type, or snack type)	Mortality	In old-old subjects≥75y.o., mortality rate was higher in greasy type to healthy (HR 3.03, 95%CI 1.07–8.57).
Silva, R. et al.[170]	Meta-analysis	Meta-analysis of 7 observational studies of community-dwelling older adults (≥60y.o.)	3.5-9years	Adherence to a Mediterranean diet (Trichopoulou index, MeDi score, etc.)	Frailty (CHS), functional disability (IADL scale, SPPB, etc.) and sarcopenia.	Higher adherence to a Mediterranean diet was inversely associated with frailty (OR 0.42, 95% CI 0.28-0.65), functional disability (OR 0.75, 0.61-0.93), but not with sarcopenia.
Kojima, G. et al.[171]	Meta-analysis	Meta-analysis of 4 observational studies of community-dwelling older adults (≥60y.o.)	3.9 years	Adherence to a Mediterranean diet (Mediterranean dietscore (MDS))	Incidence of frailty (CHS, FRAIL Scale)	Higher adherence to a Mediterranean diet was inversely associated with frailty incidence (MDS 4-5; OR 0.62, 95% CI: 0.47-0.82, MDS 6-9; OR 0.44, 95% CI: 0.31-0.64, vs MDS 0-3)
Lopez-Garcia, E et al.[173]	Prospective Cohort	8970 US women with T2DM (≥60y.o.)	22 years	Adherence to a Mediterranean diet (aMED score)	Incidence of frailty (FRAIL Scale)	HR for frailty was low in Q3(0.68, 95% CI 0.53-0.88) and Q4 (0.54, 0.42-0.71) vs Q1 in aMED score.
Wu, L. et al.[174]	Meta-analysis	Meta-analysis of 9 observational studies(≥45y.o.)	2.2-12 years	Adherence to a Mediterranean diet (Mediterranean dietscore (MDS))	Incidence of cognitive disorder (MCI, AD, dementia)	High MDS score was inversely associated with incidence of cognitive disorder (RR 0.79, 95%CI 0.70-0.90) vs low MDS score group
Valls-Pedret, C. et al.[175]	RCT	Cognitive healthy Spanish adults at high cardiovascular risk (men;55-80 years, women; 60-80 years)	4.1 years (median)	Mediterranean diet(MD) plus olive oil, MD plus nuts, or control	mean *z* scores of change 3 cognitive composites: memory, frontal, global	MD + olive oil improved frontal and global cognition, and MD + nuts improved memory vs control

Fard, N.R.P. et al.[177]	Meta-analysis	Meta-analysis of 9 observational studies(≥45y.o.)	2-12 years (longitudinal studies)	Comparison of 2 (healthy/unhealthy) ldiet patterns	Prevalence and incidence of frailty (CHS, Frailty index, etc.)	Higher adherence to a healthy diet was inversely associated with frailty (OR 0.69, 95% CI 0.57-0.84)

FFQ, **food frequency questionnaire**; CHS, Cardiovascular Health Study; SPPB, Short Physical Performance Battery; MNA, Mini Nutritional Assessment; CDT, clock drawing test; MMSE, mini-mental state examination; CANTAB, Cambridge Neuropsychological Test Automated Battery; CVLT, California Verbal Learning Test; DHA, docosahexaenoic acid; EPA, eicosapentaenoic acid; PUFA, polyunsaturated fatty acids; IADL, instrumental activities of daily livingMCI, mild cognitive impairment
